# Approaches for Targeting *Naegleria fowleri* Using Nanoparticles and Artificial Peptides

**DOI:** 10.3390/pathogens13080695

**Published:** 2024-08-16

**Authors:** Hayley Fong, Zachary H. Leid, Anjan Debnath

**Affiliations:** Center for Discovery and Innovation in Parasitic Diseases, Skaggs School of Pharmacy and Pharmaceutical Sciences, University of California San Diego, La Jolla, CA 92093, USA; hafong@ucsd.edu (H.F.); zachleid@gmail.com (Z.H.L.)

**Keywords:** *Naegleria fowleri*, primary amoebic meningoencephalitis, free-living amoeba, blood–brain barrier, nanoparticles, nanomaterials, shuttle peptide

## Abstract

*Naegleria fowleri* is a free-living amoeba which causes primary amoebic meningoencephalitis (PAM). Although PAM is rare, the fatality rate is staggering at over 97%. So, the importance of finding an effective treatment and cure for PAM caused by *N. fowleri* is a crucial area of research. Existing research on developing novel therapeutic strategies to counter *N. fowleri* infection is limited. Since the blood–brain barrier (BBB) presents an obstacle to delivering drugs to the site of infection, it is important to employ strategies that can effectively direct the therapeutics to the brain. In this regard, our review focuses on understanding the physiology and mechanisms by which molecules pass through the BBB, the current treatment options available for PAM, and the recent research conducted in the decade of 2012 to 2022 on the use of nanomaterials to enhance drug delivery. In addition, we compile research findings from other central nervous system (CNS) diseases that use shuttle peptides which allow for transport of molecules through the BBB. The approach of utilizing BBB shuttles to administer drugs through the BBB may open up new areas of drug discovery research in the field of *N. fowleri* infection.

## 1. Introduction

*Naegleria fowleri* is free-living protozoan first discovered in 1965 following reports of infection in Australia [1]. There are three distinct morphological forms the parasite can take depending on the surroundings. The first stage is a metabolically inactive cyst which can survive harsh environmental conditions [2]. Cysts can transform into the second stage known as trophozoites, which is the form in which the parasite can reproduce and infect hosts [1]. The third stage is a mobile flagellated form that is favored during times of low nutrient availability [1]. The transformations of *N. fowleri* amoeba between life stages are reversible and may not be necessary throughout the lifespan of the cell.

Of the three morphological life stages mentioned above, only the trophozoite form is infectious to humans, and amoeba must infect the host by entering through the nasal cavity [1]. Once in the nasal cavity, the trophozoites propel themselves along the olfactory nerve through locomotion via pseudopodia [3]. The trophozoites then progress to the cribriform plate—a structure with mild porosity, the most porous being found in children—and proceeds to the olfactory bulbs, thus invading the central nervous system (CNS) [3,4]. It is in the olfactory bulbs of the CNS that the presence of the trophozoites elicits an immense immune response known as primary amoebic meningoencephalitis (PAM) [3].

The symptoms of PAM start between 1 to 12 days (median 5 days) post-nasal exposure to infected water sources. Stage 1 symptoms include severe frontal headache, fever, nausea, and vomiting, before progressing to stage 2 symptoms of stiff neck, seizures, altered mental status, hallucinations, and coma [5]. Patients die between 1 and 18 days (median 5 days) following the onset of symptoms, with over 97% of cases resulting in death [5].

Despite the high fatality rate, there are only few studies performed that suggest a universally effective treatment. PAM, because of *N. fowleri* infection, is considered a “rare disease”, as defined by the U.S. Rare Diseases Act of 2002. Additionally, the short time between symptom onset and stage 2 symptoms creates difficulty in establishing and executing treatment plans. Between 2012 and 2022, there were 34 known cases of PAM in the United States.

This review will discuss some of the challenges in treating PAM and the current standard of care for infected patients. This review will also look at recent research in a growing focus on how to overcome one of the largest challenges with *N. fowleri* infection.

## 2. Challenges in Crossing the Blood–Brain Barrier (BBB)

The blood–brain barrier (BBB) presents the largest obstacle in countering *N. fowleri* infection and other neurological diseases. The BBB is a selective barrier formed of endothelial cells responsible for regulating the transfer of ions and nutrients [6]. Additionally, the extreme selectivity of the BBB serves an additional purpose of preventing neurotoxic and other foreign molecules from crossing into the brain [6].

Each component of the BBB is designed to block the entry of foreign molecules or to functionally support the other components. The activity of the BBB can be attributed to five structures: endothelial cells, astroglia (or astrocytes), pericytes, tight junctions, and adherens junctions [7]. Endothelial cells are long and flat cells that line the cerebral blood vessels, a sort of “fence” surrounding the brain. The two types of astrocytes work to regulate dynamic signaling pathways, such as those to regulate vascular function, facilitate ion homeostasis, tune brain blood flow, and balance neuroimmune responses. Pericytes are located adjacent to the endothelial cells in the basement membrane. These structures control neurovascular unit function and are critical to maintaining structural integrity of the endothelial cells, as they release signaling factors that determine the quantity of tight junctions stabilizing endothelial cells and polarizing the end feet of the astrocytes. Adherens junctions are similar to tight junctions as they are in between endothelial cells, but they also link the endothelial cells to the cytoskeleton, thus strengthening the integrity of the BBB. Tight junctions, as mentioned before, are involved in maintaining the structural integrity of the endothelial cells. Tight junctions are located between the endothelial cells and strengthen the selective permeability barrier. In a healthy patient, these components are crucial; however, due to the ability of *N. fowleri* trophozoites to invade the CNS of human hosts, the ability for drugs to infiltrate the BBB becomes critical in treatment [7].

In order to effectively design drugs and formulations that could potentially infiltrate the BBB, it is important to understand pre-devised pathways in which small molecules are able to penetrate the selectively permeable system. One of the most widely investigated methods is receptor-mediated transcytosis (RMT). In typical delivery of small molecules to the brain across the BBB, ligands bind to their cognate receptors on the luminal membrane of the brain’s microvascular and capillary endothelial cells. Through endocytosis, vesicles can surround the ligands and bring them across the membrane. Through intracellular trafficking and vesicular sorting, the ligand-containing vesicles are directed to the abluminal membrane, where it will then fuse and deliver the contents to the brain parenchyma [8]. The rate at which RMT occurs is proportional to the rate at which the endocytic vesicles partition into the early endosome fractions of the BBB [8].

By understanding the physiology and mechanisms in which molecules can pass through the BBB, specific target receptors can be identified, and carrier proteins can be conceptualized. The target receptor of interest or the carrier protein used should be one that is highly expressed in the endothelial cells lining the vasculature of the BBB; however, it must also have minimal expression in any peripheral vasculature to limit any potentially harmful peripheral effects [8].

This review will focus on therapeutic agents that can be delivered to the brain using non-invasive techniques, such as nanoparticle systems and biological mechanisms (RMT and cell-penetrating peptides), to improve the treatment options for PAM.

## 3. *Naegleria fowleri*: Pathogenesis and Diagnosis

*N. fowleri* is an opportunistic pathogen, indicating that it may infect a host should one become readily available. Due to the parasite’s affinity for warmer climates as well as their aqueous environments, most infections occur during the summer months of more southern states in the US. The most susceptible hosts for the parasites are adolescents and young adults who participate in recreational water activities. These may also occur through the use of neti pots, in which water is moved through the sinuses for the purpose of rinsing.

Infection occurs when the amoeba passes through the nasal cavity, adhering to the nasal mucosa and penetrating it, and moves along the olfactory nerve through locomotion with a pseudopod [9]. From the olfactory nerve, the trophozoites cross the cribriform plate until they reach the olfactory bulbs, thus effectively invading the central nervous system. The trophozoites at this stage of invasion can access the brain and induce an inflammatory immune response, PAM [9].

Diagnosis of PAM is performed by analysis of the cerebrospinal fluid (CSF) of the patient, obtained via lumbar puncture [10,11]. A wet mount of CSF is examined under a microscope to seek the presence of motile trophozoites. Additionally, staining CSF with Giemsa or Wright would help identify the trophozoites if present [10,11].

Both the intracranial and CSF pressures are elevated, to up to 300 to 600 mm H_2_O. The CSF can present as grey to yellowish-white and slightly discolored with red blood cells (up to 250/mm^3^) [10,11]. As the disease progresses, the color of the CSF may change due to the increasing number of red blood cells, up to 24,600/mm^3^ [10,11]. White blood cells, consisting mostly of polymorphonuclear leukocytes (PMN), can range from 300/mm^3^ to 26,000/mm^3^ [10,11].

In many reported fatalities, the cause of death is attributed to the increased intracranial pressure with brain herniation as a result of PAM, ultimately leading to cardiopulmonary arrest and pulmonary edema [10,11].

## 4. Current Treatment Protocols

In nearly 60 years of reported cases of PAM associated with *N. fowleri* infection, less than 3% of patients survived. Infection by *N. fowleri* is rare, rapid acting, and almost fatal, which makes clinical trials exceedingly difficult. The standard of care recommended by the US Centers for Disease Control and Prevention (CDC) (Table 1) is based heavily on empirical data from case reports, and the treatments were established from previous case reports or in vitro studies.

The first in vitro investigation regarding amphotericin B (AmB) for its amoebicidal activity was reported by Schuster and Rechthand in 1975 [13]. The authors used *N. fowleri* recovered from isolates of victims in Australia and the United States. It was found that a dosage of 0.075 μg/mL of AmB was required to reach the minimum growth inhibitory level in vitro. The concentrations of AmB tested, 0.25 to 1.0 μg/mL, were all found to be amoebicidal [13]. A later study by Lee et al. published in 1979 corroborated the potency of AmB through in vitro studies [14]. Lee et al. quantified the results using the minimal inhibitory concentrations (MICs) of *N. fowleri* strain HB-1 treated with compounds. For AmB in its conventional form, MIC of 0.024 μg/mL was found. This is in comparison to a methyl ester derivative of AmB which had an MIC of 0.103 μg/mL. No other compounds chosen for this study were comparable, and AmB would remain the “gold standard” for in vitro trophocidal studies [14].

The in vitro efficacy of miltefosine against *N. fowleri* was investigated by Schuster et al. (2006) due to its previously demonstrated ability to cross the BBB and accumulate within the brain tissue [15,16]. Miltefosine was developed as an anticancer drug, but was later applied to treatment of leishmaniasis and various trypanosomiases [17]. The authors evaluated the in vitro amoebicidal activity of miltefosine against *N. fowleri* strains V387, V414, and V511. For *N. fowleri* of strain V414, the MIC of miltefosine was found to be 40 μM and the minimum amoebicidal concentration (MAC) was 55 μM. However, the authors found that maintaining a concentration of 20 μM or greater of miltefosine would inhibit the growth of E6 cells (monkey kidney cells) when incubated for 2 or more days [15].

In 2013, a previously healthy 12-year-old girl was admitted to the Pediatric Intensive Care Unit following a confirmed diagnosis of infection by *N. fowleri*. The patient’s symptoms were reported to begin 7 days following exposure at an outdoor water park. Diagnosis was confirmed by the presence of trophozoites in a Giemsa–Wright scan of her cerebrospinal fluid (CSF). Initial treatment included conventional amphotericin B (intravenous, 1.5 mg/kg/day, 2 divided doses), fluconazole (intravenous/oral, 10 mg/kg/day), rifampin (intravenous/oral, 10 mg/kg/day), and azithromycin (intravenous/oral, 10 mg/kg/day). She was concurrently treated with dexamethasone (intravenous, 0.6 mg/kg/day, four divided doses), then was started on miltefosine (oral, bi- or tri-daily with 50 mg tablets) shortly after. After 55 days of hospitalization, the patient was discharged from in-patient care and was reported to have no neurological or physical deficits 6 months post-infection [18].

The validity of miltefosine as a candidate for treating PAM was reported in 2022. An 8-year-old boy was admitted to a pediatric hospital in Peru within 24 h of symptom onset. The symptoms began between 24 to 48 h post-exposure (the patient was exposed to potential infection sites on two separate occasions). Diagnosis was based on analysis of a wet mount of a nasal swab. Following a confirmed diagnosis, the patient was started on amphotericin B deoxycholate (36 mg/day, intravenous), voriconazole (220 mg every 12 h, intravenous), miltefosine (25 mg every 8 h, orally), and rifampin (360 mg/day, orally). Concurrently, the patient was treated with mannitol (54 g/day) and dexamethasone (5.4 mg/dose) to manage intracranial hypertension. Symptoms improved after 5 days with CSF showing no trophozoites after 20 days of therapy. The patient recovered with no significant changes in neurological or physical function [19].

The successful treatment of PAM is not universal amongst all patients, even when the infection is diagnosed early. The goal of future research is to find an effective and safer method toward treating *N. fowleri* infection.

## 5. Recent Research on Trophocidal Compounds

Research articles collected for this review were searched using PubMed and Google Scholar with the keywords “*Naegleria fowleri*” in combination with “blood-brain barrier”, “nanoparticles”, or “shuttle peptide”. Additional search for “blood-brain barrier” was conducted with “nanoparticles” or “shuttle peptide”. Sources for nanomaterials were constrained to the years including and between 2012 to 2022.

### 5.1. Potentially Crossing BBB

As previously mentioned, crossing the BBB presents a major obstacle in combating the onset of PAM. It is also difficult to develop in vitro assays that sufficiently mimic the BBB to ensure that the drug therapy can reach the target of interest.

Some therapeutic compounds can cross the BBB. Rifampicin, one of the standards of care for PAM, has been found in significant levels in examined CSF when given intravenously [20]. The concentration of rifampin in the CSF remained above the minimum inhibitory concentration for susceptible pathogens, such as meningeal tuberculosis, in patients with both inflamed and uninflamed meninges [21]. Fluconazole, a fungicidal azole, readily crosses the BBB and enters the CSF regardless of meningeal inflammation [22].

Phenytoin, a clinically approved drug to prevent seizures, has amoebicidal activity in vitro against *N*. *fowleri* that is comparable to the “gold standard” AmB [23]. In early animal studies, phenytoin was found to make quick entry into the brain parenchyma. A kinetic study of children (age 2 to 11 years old) treated with phenytoin showed that the concentration in the CSF was found to remain stable around 2 μg/mL for up to 24 h after administration [24]. In contrast, AmB has extremely limited ability to enter the brain—detected in low concentrations in brain tissue during postmortem studies of humans [25].

### 5.2. Sterol Biosynthesis Inhibitors

The current recommended treatment for PAM includes fluconazole, which is a sterol 14-demethylase (CYP51) inhibitor. Although fluconazole is not particularly active on *N. fowleri* and exhibits a double-digit potency in vitro, it can pass through the BBB and rapidly distribute to different CNS compartments [26]. Based on the penetration of brain tissue by fluconazole, other CYP51 inhibitors were investigated to identify more potent inhibitors. Two inhibitors in particular, itraconazole and posaconazole, were an order of magnitude more potent than AmB, while ketoconazole and isavuconazole were equipotent to AmB [26]. Although ketoconazole and isavuconazole demonstrate BBB penetration in animal models, the highly active inhibitors itraconazole and posaconazole produce low drug concentrations in CNS specimens [27,28,29,30]. Therefore, identification of potent and brain-penetrant CYP51 inhibitors may serve as predecessor for future anti-PAM therapeutics.

Apart from CYP51, sterol C24-methyltransferase (SMT), sterol Δ^8^−Δ^7^ isomerase (ERG2), protein farnesyltransferase (FT), and HMG-CoA reductase are involved in the synthesis of ergosterol in *N. fowleri*. While FDA-approved inhibitors of SMT, ERG2 and FT, abafungin, tamoxifen, and lonafarnib displayed a single-digit micromolar potency, the HMG-CoA reductase inhibitors fluvastatin and pitavastatin exhibited nanomolar potency against *N. fowleri* [31,32,33]. BBB permeability of some of these inhibitors warrants further evaluation to confirm their efficacy in vivo.

### 5.3. Nanomaterials Demonstrating Efficacy In Vitro

The use of nanomaterials in medicine is a rapidly growing field with a multitude of possibilities. Some therapeutic agents when conjugated to a nanomaterial display greater chemical and biological stability and can allow for controlled release of a drug—an advantage when attempting to limit the frequency of dosing or the concentration of the dose. Nanomaterials can be tuned to have various surface ligands such as proteins and antibodies, thus potentially binding to a receptor on a target of interest. The surface area within the nanomaterial can also allow for carrying a high drug load within a small space (between 10 and 1000 nm), which is advantageous when trying to transport very large molecules or proteins through a small pore [34].

This review will focus on polymeric nanoparticles, which are constituted of a repeating motif. The nanoparticles developed for combating *N. fowleri* infection are structured around metal centers such as silver or gold. However, organic polymeric nanomaterials such as poly-lactic-co-glycolic-acid (PLGA) are useful due to their high biocompatibility and biodegradability [34]. The studies reported in this section are summarized in Table 2.

#### 5.3.1. Silver

The use of silver nanomaterials in medicine is a growing field due to their antimicrobial properties and tunability—the mechanism by which silver nanoparticles (AgNPs) can be altered by changing their size, shape, surface charge, concentration, and colloidal state [35]. In this section, the use of drug-conjugated AgNPs for the purpose of combating *N. fowleri* and another free-living amoebic infection is discussed.

##### AmB and Nystatin Conjugated to Silver Nanoparticles

Rajendran et al. (2017) [36] reported the use of a silver nanoparticle conjugated with AmB, nystatin (Nys), and fluconazole (Flu), with a silver-to-drug ratio of 4:1 and 1:1. The amoebicidal activity of the AgNPs was tested against *N. fowleri* ATCC 30174 and the positive control of AmB alone. The effects of the AgNPs and drugs alone were also tested to eliminate any unilateral amoebicidal activity. Trophozoites were incubated with varied concentrations of compounds and controls for 24 h at 37 °C.

At 2.5 μM of AmB-AgNP, trophozoite viability was reduced from 9.2 × 10^5^ to 2 × 10^5^, compared to AmB alone with a reduction to 3.7 × 10^5^. The other two drug-conjugated nanoparticles were not as effective: Nys and Nys-AgNP reduced trophozoite viability to 7.5 × 10^5^ and 5.8 × 10^5^, respectively. Flu-AgNPs had limited amoebicidal activity, and the treatment with the silver nanoparticles alone demonstrated no activity.

Regarding host cell cytotoxicity, HeLa (Henrietta Lacks) cervical cancer cells were used. When *N. fowleri* trophozoites and HeLa cells are co-incubated, host cell damage is caused (75%). In contrast, trophozoites pre-treated with the drug-conjugated AgNPs were able to reduce the amount of damage to the host cells. At 2.5 μM of AmB-AgNP, less than 10% of host cells were damaged, compared to AmB alone with approximately 20% host cell damage. The authors posit that the effects of the drugs are enhanced in the nanoconjugate form due to the small size and surface properties [36].

##### Flavonoid-Conjugated Nanoparticles Stabilized by Plant Gums

Following the success of the AmB-AgNPs reported in 2017, the same authors investigated if metal nanoparticles coated with natural compounds could also have amoebicidal properties [37]. They looked at the flavonoids hesperidin (HDN) and naringin (NRG). The use of flavonoids as therapeutic agents is limited due to their poor solubility and propensity to undergo chemical changes due to environmental conditions. This was overcome by the use of plant gums: gum tragacanth (GT) and gum acacia (GA). GA, in particular, was chosen due to it already being used by food and pharmaceutical companies as a stabilizer and controlled release agent [38,39].

A variety of component combinations were tested against *N. fowleri* trophozoites (ATCC 30174), and the results were compared against amoeba alone and AmB alone. At 25 μg/mL, GA-AgNPs-HDN reduced trophozoite viability by 99%, which was significantly more than treatment with AmB at either 25 or 50 μg/mL. Treatment with 25 or 50 μg/mL of GA alone, HDN alone, or AgNP alone did not surpass efficacy of AmB [37].

The amoebicidal activity of various doses of these materials was also determined for trophozoites of another free-living amoeba, *Acanthamoeba castellanii* (ATCC 50492). The relative activity was compared against amoeba alone and chlorhexidine. A dose of 50 μg/mL GA-AgNP-HDN eliminated all trophocidal activity, and reducing the concentration to 25 μg/mL of this material reduced trophozoite growth by 90%. This showed greater efficacy than the chlorhexidine control, and significantly more activity than the components themselves [37].

When GA-AgNPs-HDN was evaluated for cytotoxicity against HeLa cells, the nanocomposite only exhibited 11% cytotoxicity at 100 μg/mL. GT-AuNPs-NRG exhibited 23% cytotoxicity toward HeLa cells, but due to its relatively low amoebicidal activity against *N. fowleri*, its therapeutic potential is limited. The individual components of GA, HDN, and AgNPs all produced less than 50% cytotoxicity against HeLa cells when dosed at 100 μg/mL [37].

##### Guanabenz Silver- and Gold-Conjugated Nanoparticles

In the same interest of naturally derived compounds, Anwar et al. (2019) explored the potential of guanabenz (GNB) as an antiparasitic agent in conjugation with silver and gold nanoparticles [40]. GNB is an FDA-approved drug therapy for the treatment of hypertension. The authors had previously reported the efficacy of GNB against *Toxoplasma gondii* and *Plasmodium falciparum* [41,42]. GNB was found to reduce neuroinflammation and associated symptoms in mice infected with the brain-infective *T. gondii* parasites [42].

The amoebicidal efficacy was compared to treatment with 100 μM of AmB; the positive control reduced the trophozoite viability from approximately 3.5 × 10^5^ to 2.5 × 10^5^. Treatments of amoeba with only gold and silver were ineffective, whereas 50 μM and 100 μM of GNB were both more effective at reducing cell viability than AmB. The gold-conjugated and silver-conjugated GNB nanoparticles both demonstrated amoebicidal activity greater than AmB at a concentration as low as 2.5 μM [40].

The authors also demonstrated the potential use for GNB-AgNP and GNB-AuNPs with regard to *A. castellanii* genotype T4 trophozoites (ATCC 50492). The efficacy was compared to 100 μM of chlorhexidine, which reduced cell viability from 5.9 × 10^5^ to nearly 0. Treatment of trophozoites with 2.5 μM GNB alone reduced cell viability by approximately 15%; the same concentration of silver showed a similar effect. In contrast, 2.5 μM GNB-AgNP had significantly greater amoebicidal effect at approximately 83% [40].

HeLa cells experienced limited toxicity due to treatment with GNB, silver or gold, or the GNB nanoparticles. At the highest dose of 100 μM, GNB alone exhibited approximately 30% toxicity against human keratinocyte skin (HaCat) cells. The overall toxicity of the GNB nanoconjugates never exceeded 20% toxicity toward either human cell line [40].

#### 5.3.2. Gold

Gold nanoparticles (AuNPs) are tunable for various purposes in biomedical applications such as bioimaging. Typically, AuNPs are surface functionalized for the materials to target specific disease sites. Various functional groups and ligands can be attached to the surface of the nanoparticles, such as proteins or antibodies [43].

Many of the previously mentioned drug-conjugated nanoparticles included an investigation of using both gold and silver, but none of the drugs conjugated to gold had particularly greater amoebicidal activity than their silver counterparts. The nanomaterials discussed in this section were exclusive in their study of drugs conjugated to gold centers only.

##### *Trans*-Cinnamic Acid-Conjugated Gold Nanoparticles (CA-AuNPs)

Cinnamic acid (CA) was isolated from plant matter and studied for its antimicrobial activities. When used alone, CA significantly reduced the cell viability of *N. fowleri* trophozoites (clinical strain ATCC 30174) at concentrations as low as 2.5 μM, although this was not as effective as treatment with 50 μM of AmB. In comparison, an equal concentration of the CA-AuNP was more effective and reduced the trophozoite viability two-fold. At a high concentration of 50 μM CA-AuNPs, the amoebicidal activity was similar to that of an equal concentration of AmB, reducing the cell viability from 2.6 × 10^5^ to 1.9 × 10^5^ (compared to AmB with a reduction to 1.6 × 10^5^) [44].

When trophozoites were pre-treated with CA and CA-AuNPs, the authors observed inhibition of amoeba-mediated host cell damage toward HeLa cells, demonstrating potential to reduce the cell invasion of *N. fowleri*. There were no observed cytotoxic effects of CA and CA-AuNPs toward HeLa cells [44].

##### Curcumin-Conjugated Gold Nanoparticles

Curcumin is the active component of turmeric, medicinally understood for its anti-inflammatory and antioxidant properties. The polyphenol was previously shown to have inhibitory activity against fungal and parasitic infections at micromolar concentrations and can be safely tolerated by humans in large doses [45].

Curcumin was incubated with *N. fowleri* trophozoites (ATCC 30174) at 6.25, 12.5, 25, and 200 μM for 24 h at 37 °C. At 200 μM, curcumin exhibited 66% amoebicidal activity. The remaining doses of 6.25, 12.5, and 25 μM each displayed no more than 35% amoebicidal activity. The IC_50_ of curcumin with *N. fowleri* trophozoites was calculated to be 74 μM. Comparatively, only 10 μM of curcumin-AuNP was required to produce 69% amoebicidal activity. Equal concentrations of gold nanoparticles and curcumin alone exhibited 0 and 32% amoebicidal activity, respectively [45].

The authors also tested these nanoparticles against another free-living amoeba, *Balamuthia mandrillaris* (ATCC 50209). Dosages of 50 and 200 μM curcumin displayed 34% and 55% amoebicidal activity, respectively. The calculated IC_50_ of curcumin with *B. mandrillaris* was 172 μM. When conjugated with AuNP, the cell viability of *B. mandrillaris* was reduced to 78% with a dosage as low as 10 μM. The same concentration of AuNP and curcumin did not significantly inhibit cell growth [45].

Cytotoxicity toward human cell lines was evaluated using HeLa and HaCaT cells. At concentrations below 10 μM, curcumin had no cytotoxic effects toward either human cell lines. Only 2% and 8% cytotoxicity levels were observed at 12.5 and 50 μM concentrations, respectively. Neither the AuNPs nor curcumin-AuNPs produced any significant cytotoxicity [45].

#### 5.3.3. Other Nanomaterial Conjugates

##### Polyaniline-Hexagonal Boron Nitride (PANI/hBN)

While boron nitride (BN) is an artificial material (not found in nature), it is incredibly stable and exists in numerous forms, such as cubic and hexagonal. Abdelnasir et al. synthesized a nanocomposite of hexagonal BN (hBN) within a conducting polymer matrix of polyaniline (PANI) due to the literature precedent of both individual components being biocompatible and demonstrating antimicrobial properties [6]. The nanocomposite was formed at different ratios of PANI:hBN (1:1, 1:2, and 1:5), and the various ratios were tested for host cell toxicity and amoebicidal activity against *N. fowleri* trophozoites. The results of the cysticidal activity against *N. fowleri* and activity against trophozoites and cysts of *A. castellanii* are mentioned in the study [46].

The three ratios of PANI/hBN nanocomposites—1:1, 1:2, and 1:5—were dosed at 100 μg/mL against *N. fowleri* trophozoites (HB-1). The amount of BN and PANI that corresponds to the three ratios was used as a control to account for any inherent amoebicidal activity of the components. At 100 μg/mL of PANI/hBN at 1:5, trophozoite growth was inhibited by 58%. The same dose of the 1:1 nanocomposite reduced trophozoite growth by 50%, and the 1:2 nanomaterial was only able to reduce cell viability by 40%. By comparison to the controls, PANI and BN, only the 1:1 and 1:5 ratios demonstrated a significant reduction in cell viability [46].

The authors also tested these PANI/hBN nanomaterials against *A. castellanii* trophozoites of the genotype T4 (ATCC 50492). A quantity of 100 μg/mL of PANI/hBN at 1:5 reduced the cell viability of *A. castellanii* trophozoites by 55%. The 1:1 and 1:2 nanocomposites reduced cell viability by 35% and 47%, respectively. There were no significant reductions in cell viability when treated with either PANI or BN alone [46]. 

Host cell toxicity of the PANI/hBN materials was determined using primary human corneal epithelial cells (pHCECs) and HaCaT cells. At 100 μg/mL of PANI/hBN, 100 μg/mL of PANI alone, and 500 μg/mL of BN alone, minimal toxicity was found toward pHCECs and HaCaT cell lines. The three ratios of PANI/hBN (1:1, 1:2, and 1:5) at 100 μg/mL displayed 15, 0, and 12% cytotoxicity toward HaCaT cells, respectively, and even lower cytotoxicity toward pHCECs [46].

##### Polyaniline: Tungsten Disulfide (PANI:WS_2_) Nanoparticles

Tungsten disulfide (WS_2_) belongs to a class of nanomaterials called transition metal dichalcogenides (TMDCs), which are graphene-like two-dimensional materials that produce weak van der Waal forces but strong covalent bonding between layers. WS_2_ nanomaterials, in particular, have been reported to have antibacterial activity and ROS-independent mechanisms [47,48]. The authors synthesized PANI:WS_2_ nanocomposites for a study of their action against *N. fowleri* and *A. castellanii* [49].

Nanocomposites were synthesized to generate three ratios of PANI:WS_2_: 1:1, 1:2, and 1:5. *N. fowleri* trophozoites were dosed at 100 μg/mL for initial assessment of amoebicidal activity, with comparison against untreated trophozoites and miltefosine-treated trophozoites. At a 1:1 ratio of PANI:WS_2_, trophozoite viability was reduced by 20%. The 1:2 and 1:5 nanocomposites were more active at reducing trophozoite viability, with 36% and 54% reductions, respectively. Still, the nanocomposites showed better amoebicidal activity than the WS_2_ and PANI components alone [49].

*A. castellanii* trophozoites of T4 genotype (ATCC 50492) were also subject to treatment with PANI:WS_2_ by Abdelnasir et al. [49]. Amoebae were incubated with 100 μg of 1:1, 1:2, and 1:5 ratios of PANI:WS_2_ nanocomposites. Results were compared against amoebae alone and chlorhexidine. The 1:1 ratio gave 33% growth inhibition, and both the 1:2 and 1:5 ratios gave 48% growth inhibition. The components, PANI and WS_2_, alone gave only 20% and 23% growth inhibition, respectively [49].

Host cell toxicity was evaluated using pHCEC and HaCaT cell lines at equal concentrations used to measure amoebicidal activity. PANI:WS_2_ (1:1) exhibited a maximum toxicity of 11% toward HaCaT cells, with 1:2 and 1:5 composites having 6% and 0% cell death, respectively. Both WS_2_ and PANI caused less than 10% cell death toward HaCaT cells, compared to miltefosine causing 46% cell death toward the same cell line [49].

**Table 2:** In vitro efficacy of drug-conjugated nanomaterials against *N. fowleri* trophozoites.

Compounds	Average Diameter of Nanomaterials (nm) ^1^	Result of In Vitro Studies ^2^	Reference
AmB-AgNPs	10–90	Trophozoites were treated with compounds at 2.5 μM for 24 h. AmB-AgNP-treated trophozoites saw amoeba viability reduced to 22% compared to AmB-only treated trophozoites at 40% viability.	[36]
Nystatin-AgNPs	10–90	Trophozoites were treated with compounds at 2.5 μM. Nystatin alone reduced trophozoite viability to 82% compared to nystatin-AgNPs, which further reduced cell viability to 63%.	[36]
GA-AgNPs-HDN	182.8	Trophozoites were incubated with compounds at 25 μg/mL.	[37]
		GA-AgNPs-HDN reduced trophozoite viability by 99% and was more effective than cell treatment with AmB alone.	
GNB-AgNPs GNB-AuNPs	50–150 50–70	Trophozoites were incubated with compounds at 2.5 and 5 μM. The amoebicidal activity of the GNB-conjugated nanoconjugates was compared against trophozoites treated with 100 μM of AmB. GNB alone only displayed anti-amoebic activity at 50 and 100 μM, whereas the GNB-conjugated nanoconjugates displayed high potency as low as 2.5 μM.	[40]
CA-AuNPs	89	Trophozoites were treated with varying concentrations of compounds, and the amoebicidal activity was compared to 50 μM AmB. Treatment with 50 μM AmB reduced trophozoite viability from approximately 9.5 × 10^5^ to 1.6 × 10^5^, which is comparable to the anti-amoebic effect shown for the CA-AuNPs.	[44]
Curcumin-AuNPs	53	Trophozoites were treated with varying concentrations of compounds. Amoebicidal activity was compared against miltefosine. At 200 μM, curcumin alone displayed 66% amoebicidal activity against *N. fowleri* trophozoites, and the IC_50_ was determined to be 74 μM. Comparatively, 10 μM of curcumin-AuNPs displayed 69% amoebicidal activity. AuNP alone showed insignificant amoebicidal activity.	[45]
PANI/hBN	N.D.	Trophozoites were treated with 100 μg/mL of PANI/hBN nanocomposite ratios of 1:1, 1:2, and 1:5. Amoebicidal activity was compared against miltefosine. Treatment with 1:5 PANI/hBN reduced cell viability by 58%. The 1:1 and 1:2 PANI/hBN treatments only inhibited trophozoite growth by 50% and 40%, respectively. The individual components were less effective at reducing trophozoite viability.	[46]
PANI-WS_2_	N.D.	Trophozoites were treated with 100 μg/mL of 1:1, 1:2, and 1:5 PANI-WS_2_ nanocomposites. Amoebicidal activity was compared against miltefosine. The 1:5 PANI-WS_2_ nanocomposite was the most effective at reducing trophozoite viability, by 54%, followed by 1:2 with 36% and 1:1 with 20%. WS_2_ and PANI alone were less effective at reducing cell viability.	[49]

N.D., not determined. ^1^ Nanoparticle size was determined through atomic force microscopy. ^2^ In vitro studies were conducted against *N. fowleri* strain HB-1 (ATCC 30174) unless otherwise indicated. Drug-treated trophozoites were incubated for 24 h at 37 °C.

### 5.4. Shuttle Peptide

Another approach to developing carriers that can direct drug administration through the BBB is a molecular vector, or BBB shuttles. These are also referred to as brain-permeable peptide-drug conjugates (PDCs) or BBB-targeting ligands [50]. This construct was inspired by the use of chimeric proteins to target cell receptors, allowing for the transport of small molecules, proteins, nanoparticles, and genetic material across the BBB [51]. The use of synthetic peptides to construct these shuttles is of much interest because of their chemical and biological advantages: they are of low cost but highly specific, easy to characterize and synthesize but with low immunogenicity, and easily tunable. Because of the versatility of peptide design, the drug of interest is less likely to be altered in order to fit the carrier [51].

The ideal BBB shuttle should target a receptor on the BBB capable to mediate transcytosis and would recognize a broad range of substrates [51]. Once the BBB shuttle is bound to a receptor, transcytosis is triggered and the therapeutic agent released and transported to the abluminal surface [50].

With countless possibilities of peptides of varying size and functionalizations, experimental methods have been developed to systematically elucidate structural advantages. Phage display technology (PDT) was developed to link a polypeptide’s phenotype (specifically binding to a target of interest) and its corresponding genotype [50]. Polypeptide sequences can be inserted into the capsid protein of a phage genome and be expressed on the surface of the bacteriophage; this can be done with many different exogenous peptide sequences [50]. Through a process called biopanning, the most effective polypeptide sequences can be identified through affinity selection to an immobilized target and sequencing of the phage DNA [50].

Díaz-Perlas et al. reported a brain-penetrating peptide drug conjugate that has in vitro potential to penetrate the BBB. The dodecapeptide SGVYKVAYDWQH (abbreviated to SGV) was developed using phage PDT as described above [52]. The resulting polypeptide underwent investigation for permeability across a selective membrane of endothelial-type cells intended to mimic the BBB and the result was compared against a pre-established BBB shuttle known as MiniAp4 [52]. The apparent permeability of SGV was 4.4 ± 0.6 × 10^−^^6^ cm/s—the same order of magnitude as MiniAp4 (6.7 ± 0.6 × 10^−^^6^ cm/s) [52]. The polypeptide exhibited low cytotoxicity toward bEnd.3 and HeLa cells when incubated for 24 h at 33.3 and 100 μM concentrations [52].

While this technology was not found to be used toward *N. fowleri* infection, the use of shuttle peptides would rapidly expand the list of potential hits, in terms of both quantity and diversity.

## 6. Conclusions

In this review, we provided an overview of *N. fowleri* as a parasitic protozoan and its infection in humans. One of the greatest challenges in treating primary amoebic meningoencephalitis is directing drug delivery across the blood–brain barrier, in addition to finding potent therapeutic agents that are rapidly acting with low toxicity.

Recently, the idea of intranasal drug delivery has been raised within the scope of PAM. Siddiqui and Khan posed the possibility of using a nebulizer that could aerosolize anti-amoebic drugs, such as amphotericin B, and target delivery to the brain [53]. There is documented use of liposomal amphotericin B solution as a nasal irrigant for pediatric mucormycosis treatment [54]. Intranasal delivery with an enolase inhibitor has been tested in an animal model of PAM [55]. In the case that intranasal delivery is feasible, compounds would be able to overcome the impermeability of the BBB which could, in turn, reduce the administered dosage to attain the MIC [53]. This route would also circumvent the hepatic first-pass metabolism of the drug [56].

One of the major limitations of intranasal drug delivery is formulation. Drugs developed for the purpose of intranasal administration need to be water-based and capable of being aerosolized for inhalation [57]. The droplet distribution and deposition of the nasal spray is also dependent on the size of the particles, viscosity, and diffusion time [57]. Nanoparticles, while highly tunable and able to carry high drug loads, may exhibit cytotoxic effects due to surface modifications and be degraded by nasal enzymes [58]. Nevertheless, this concept remains largely unexplored for feasibility and validity.

There are several intranasal peptide drugs that have been approved by the FDA or are in clinical trials, such as Nasulin^®^ and Syntocinon^®^ [59]. Nasulin^®^, the intranasal form of insulin, has undergone various formulation changes to enhance the peptide’s absorption in the nasal mucosa [59]. To evaluate this in a disease model of PAM, the amoebicidal effect of the formulation components would need to be determined.

We reviewed the reported literature in the decade of 2012–2022 that sought to develop drug-conjugated nanomaterials that were comparable to the in vitro activity of the already established standards of care. We introduced a new field that has not yet been explored within the context of *N. fowleri*.

## Figures and Tables

**Table 1:** Standards of care for treating primary amoebic meningoencephalitis.

Compound	Route of Administration and Dosage Information	Reference
Amphotericin B	Intravenous Day 1–3: 1.5 mg/kg/day in 2 divided doses Day 4–14: 1 mg/kg/day once daily OR Intrathecal Day 1–2: 1.5 mg once daily Day 3–0: 1 mg/day every other day	[12]
Miltefosine	Oral Weight < 45 kg, 50 mg twice daily Weight > 45 kg, 50 mg thrice daily Duration: 28 days	[12]
Fluconazole	Intravenous or oral 10 mg/kg/day once daily Duration: 28 days	[12]
Rifampicin	Intravenous or oral 10 mg/kg/day once daily Duration: 28 days	[12]
Azithromycin	Intravenous or oral 10 mg/kg/day once daily Duration: 28 days	[12]
Dexamethasone	Intravenous 0.6 mg/kg/day in 4 divided doses Duration: 4 days	[12]

## Data Availability

No new data were created or analyzed in this study. Data sharing is not applicable to this article.

## References

[1] Marciano-Cabral F., Cabral G.A. (2007). The immune response to *Naegleria fowleri* amebae and pathogenesis of infection. FEMS Immunol. Med. Microbiol..

[2] Güémez A., García E. (2021). Primary Amoebic Meningoencephalitis by *Naegleria fowleri*: Pathogenesis and Treatments. Biomolecules.

[3] Grace E., Asbill S., Virga K. (2015). *Naegleria fowleri*: Pathogenesis: Diagnosis, and Treatment Options. Antimicrob. Agents Chemother..

[4] Baig A.M. (2016). Primary Amoebic Meningoencephalitis: Neurocehmotaxis and Neurotropic Preferences of *Naegleria fowleri*. ACS Chem. Neurosci..

[5] Illness and Symptoms|*Naegleria fowleri*|CDC. https://www.cdc.gov/parasites/naegleria/illness.html.

[6] Lombardo S.M., Schneider M., Türeli A.E., Günday Türeli N. (2020). Key for crossing the BBB with nanoparticles: The rational design. Beilstein J. Nanotechnol..

[7] Wu D., Chen Q., Chen X., Han F., Chen Z., Wang Y. (2023). The Blood-Brain Barrier: Structure, Regulation, and Drug Delivery. Signal Transduct. Target. Ther..

[8] Terstappen G.C., Meyer A.H., Bell R.D., Zhang W. (2021). Strategies for Delivering Therapeutics across the Blood-Brain Barrier. Nat. Rev. Drug Discov..

[9] Martínez-Castillo M., Cárdenas-Zúñiga R., Coronado-Velázquez D., Debnath A., Serrano-Luna J., Shibayama M. (2016). *Naegleria fowleri* after 50 years: Is it a neglected pathogen?. J. Med. Microbiol..

[10] Martinez A.J. (1985). Free-Living Amebas: Natural History, Prevention, Diagnosis, Pathology, and Treatment of Disease.

[11] Visvesvara G.S., Moura H., Schuster F.L. (2007). Pathogenic and Opportunistic Free-Living Amoebae: *Acanthamoeba* spp.; *Balamuthia mandrillaris*, *Naegleria fowleri*, and *Sappinia diploidea*. FEMS Immunol. Med. Microbiol..

[12] Treatment|*Naegleria fowleri*|CDC. https://www.cdc.gov/parasites/naegleria/treatment-hcp.html.

[13] Schuster F.L., Rechthand E. (1975). In Vitro Effects of Amphotericin B on Growth and Ultrastructure of the Amoeboflagellates *Naegleria gruberi* and *Naegleria fowleri*. Antimicrob. Agents Chemother..

[14] Lee K.K., Karr S.L., Wong M.M., Hoeprich P.D. (1979). In Vitro Susceptibilities of *Naegleria fowleri* Strain HB-1 to Selected Antimicrobial Agents, Singly and in Combination. Antimicrob. Agents Chemother..

[15] Schuster F.L., Guglielmo B.J., Visvesvara G.S. (2006). In-Vitro Activity of Miltefosine and Voriconazole on Clinical Isolates of Free-Living Amebas: *Balamuthia mandrillaris*, *Acanthamoeba* spp., and *Naegleria fowleri*. J. Eukaryot. Microbiol..

[16] Marschner N., Kotting J., Eibl H., Unger C. (1992). Distribution of hexadecyl phosphocholine and octadecyl-methyl-glycero-3-phosphocholine in rat tissues during steady-state treatment. Cancer Chemother. Pharmacol..

[17] Sundar S., Jha T.K., Thakur C.P., Engel J., Sindermann H., Fischer C., Junge K., Bryceson A., Berman J. (2002). Oral miltefosine for Indian visceral leishmaniasis. N. Eng. J. Med..

[18] Linam W.M., Ahmed M., Cope J.R., Chu C., Visvesvara G.S., Da Silva A.J., Qvarnstrom Y., Green J. (2015). Successful Treatment of an Adolescent with *Naegleria fowleri* Primary Amebic Meningoencephalitis. Pediatrics.

[19] Martínez D.Y., Bravo-Cossio F., Valdivia-Tapia M.D.C., Carreazo N.Y., Cabello-Vilchez A.M. (2022). Successful Treatment of Primary Amoebic Meningoencephalitis Using a Novel Therapeutic Regimen Including Miltefosine and Voriconazole. Acta Parasit..

[20] Nau R., Prange H.W., Menck S., Kolenda H., Visser K., Seydel J.K. (1992). Penetration of rifampicin into the cerebrospinal fluid of adults with uninflamed meninges. J. Antimicrob. Chemother..

[21] Kaojarern S., Supmonchai K., Phuapradit P., Mokkavesa C., Krittiyanunt S. (1991). Effect of steroids on cerebrospinal fluid penetration of antituberculous drugs in tuberculous meningitis. Clin. Pharmacol. Ther..

[22] Schwartz S., Thiel E. (2009). Cerebral aspergillosis: Tissue penetration is the key. Med. Mycol..

[23] Anwar A., Rajendran K., Siddiqui R., Shah M.R., Khan N.A. (2019). Clinically Approved Drugs against CNS Diseases as Potential Therapeutic Agents to Target Brain-Eating Amoebae. ACS Chem. Neurosci..

[24] Koren G., Barzilay Z., Schachar E., Brand N., Danee S., Halkin H., MacLeod S.M. (1983). Kinetics of CSF Phenytoin in Children. Can. J. Neurol. Sci..

[25] Felton T., Troke P.F., Hope W.W. (2014). Tissue penetration of antifungal agents. Clin. Microbiol. Rev..

[26] Debnath A., Calvet C.M., Jennings G., Zhou W., Aksenov A., Luth M.R., Abagyan R., Nes W.D., McKerrow J.H., Podust L.M. (2017). CYP51 is an essential drug target for the treatment of primary amoebic meningoencephalitis (PAM). PLoS Negl. Trop. Dis..

[27] Perfect J.R., Durack D.T. (1985). Penetration of imidazoles and triazoles into cerebrospinal fluid of rabbits. J. Antimicrob. Chemother..

[28] Schmitt-Hoffmann A.-H., Kato K., Townsend R., Potchoiba M.J., Hope W.W., Andes D., Spickermann J., Schneidkraut M.J. (2017). Tissue Distribution and Elimination of Isavuconazole following Single and Repeat Oral-Dose Administration of Isavuconazonium Sulfate to Rats. Antimicrob. Agents Chemother..

[29] Sorensen K.N., Sobel R.A., Clemons K.V., Pappagianis D., Stevens D.A., Williams P.L. (2000). Comparison of fluconazole and itraconazole in a rabbit model of coccidioidal meningitis. Antimicrob. Agents Chemother..

[30] Calcagno A., Baietto L., De Rosa F.G., Tettoni M.C., Libanore V., Bertucci R., D’Avolio A., Di Perri G. (2011). Posaconazole cerebrospinal concentrations in an HIV-infected patient with brain mucormycosis. J. Antimicrob. Chemother..

[31] Zhou W., Debnath A., Jennings G., Hahn H.J., Vanderloop B.H., Chaudhuri M., Nes W.D., Podust L.M. (2018). Enzymatic chokepoints in the sterol biosynthesis pathway of *Naegleria fowleri*. PLoS Pathog..

[32] Hahn H.J., Debnath A. (2020). In Vitro Evaluation of Farnesyltransferase Inhibitor and its Effect in Combination with 3-Hydroxy-3-Methyl-Glutaryl-CoA Reductase Inhibitor against *Naegleria fowleri*. Pathogens.

[33] Hahn H.J., Abagyan R., Podust L.M., Roy S., Ali I.K.M., Debnath A. (2020). HMG-CoA Reductase Inhibitors as Drug Leads against *Naegleria fowleri*. ACS Chem. Neurosci..

[34] Joseph S.K., Arya M.A., Thomas S., Nair S.C. (2022). Nanomedicine as a future therapeutic approach for treating meningitis. J. Drug Sci. Technol..

[35] Dakal T.C., Kumar A., Majumdar R.S., Yadav V. (2016). Mechanistic Basis of Antimicrobial Actions of Silver Nanoparticles. Front. Microbiol..

[36] Rajendran K., Anwar A., Khan N.A., Siddiqui R. (2017). Brain-Eating Amoebae: Silver Nanoparticle Conjugation Enhanced Efficacy of Anti-Amoebic Drugs against *Naegleria fowleri*. ACS Chem. Neurosci..

[37] Anwar A., Masri A., Rao K., Rajendran K., Khan N.A., Shah M.R., Siddiqui R. (2019). Antimicrobial activities of green synthesized gums-stabilized nanoparticles loaded with flavonoids. Sci. Rep..

[38] Mohan Y.M., Raju K.M., Sambasivudu K., Singh S., Sreedhar B. (2007). Preparation of acacia-stabilized silver nanoparticles: A green approach. J. Appl. Polym. Sci..

[39] Benfattoum K., Haddadine N., Bouslah N., Benaboura A., Maincent P., Barillé R., Sapin-Minet A., El-Shall S. (2018). Formulation characterization and *in vitro* evaluation of acacia gum-calcium alginate beads for oral drug delivery systems. Polym. Adv. Technol..

[40] Anwar A., Mungroo M.R., Anwar A., Sullivan W.J., Khan N.A., Siddiqui R. (2019). Repositioning of Guanabenz in Conjugation with Gold and Silver Nanoparticles against Pathogenic Amoebae *Acanthamoeba castellanii* and *Naegleria fowleri*. ACS Infect. Dis..

[41] Benmerzouga I., Checkley L.A., Ferdig M.T., Arrizabalaga G., Wek R.C., Sullivan W.J. (2015). Guanabenz repurposed as an antiparasitic with activity against acute and latent toxoplasmosis. Antimicrob. Agents Chemother..

[42] Martynowicz J., Augusto L., Wek R.C., Boehm S.L., Sullivan W.J. (2019). Guanabenz Reverses a Key Behavioral Change Caused by Latent Toxoplasmosis in Mice by Reducing Neuroinflammation. mBio.

[43] Daraee H., Eatemadi A., Abbasi E., Aval S.F., Kouhi M., Akbarzadeh A. (2016). Application of gold nanoparticles in biomedical and drug delivery. Artif. Cells Nanomed. Biotechnol..

[44] Rajendran K., Anwar A., Khan N.A., Shah M.R., Siddiqui R. (2019). *trans*-Cinnamic Acid Conjugated Gold Nanoparticles as Potent Therapeutics against Brain-Eating Amoeba *Naegleria fowleri*. ACS Chem. Neurosci..

[45] Mungroo M.R., Anwar A., Khan N.A., Siddiqui R. (2020). Gold-Conjugated Curcumin as a Novel Therapeutic Agent against Brain-Eating Amoebae. ACS Omega.

[46] Abdelnasir S., Mungroo M.R., Shahabuddin S., Siddiqui R., Khan N.A., Anwar A. (2021). Polyaniline-Conjugated Boron Nitride Nanoparticles Exhibiting Potent Effects against Pathogenic Brain-Eating Amoebae. ACS Chem. Neurosci..

[47] Shang E., Niu J., Li Y., Zhou Y., Crittenden J.C. (2017). Comparative toxicity of Cd, Mo, and W sulphide nanomaterials toward *E. coli* under UV irradiation. Environ. Pollut..

[48] Agarwal V., Chatterjee K. (2018). Recent advances in the field of transition metal dichalcogenides for biomedical applications. Nanoscale.

[49] Abdelnasir S., Mungroo M.R., Shahabuddin S., Siddiqui R., Khan N.A., Ahmad I., Anwar A. (2022). Polyaniline (PANI)-conjugated tungsten disulphide (WS_2_) nanoparticles as potential therapeutic agents against brain-eating amoebae. Appl. Microbiol. Biotechnol..

[50] Zhou X., Smith Q.R., Liu X. (2021). Brain penetrating peptides and peptide-drug conjugates to overcome the blood-brain barrier and target CNS diseases. WIREs Nanomed. Nanobiotechnol..

[51] Oller-Salvia B., Sánchez-Navarro M., Giralt E., Teixidó M. (2016). Blood-brain barrier shuttle peptides: An emerging paradigm for brain delivery. Chem. Soc. Rev..

[52] Díaz-Perlas C., Sánchez-Navarro M., Oller-Salvia B., Moreno M., Teixidó M., Giralt E. (2017). Phage display as a tool to discover blood-brain barrier (BBB)-shuttle peptides: Panning against a human BBB cellular model. Pept. Sci..

[53] Siddiqui R., Khan N.A. (2023). Intranasal Route for the Delivery of Antiamebic Drugs Against brain-eating Amoeba. Ther. Deliv..

[54] Khafagy R., Gupta S., Campisi P., Waters V. (2020). Treatment of localized mucormycosis using nasal amphotericin B irrigation in pediatric oncology. Pediat. Blood Cancer.

[55] Milanes J.E., Yan V.C., Pham C.-D., Muller F., Kwain S., Rees K.C., Dominy B.N., Whitehead D.C., Millward S.W., Bolejack M. (2024). Enolase inhibitors as therapeutic leads for *Naegleria fowleri* infection. PLoS Pathog..

[56] Xu D., Song X.-J., Chen X., Wang J.-W., Cui Y.-L. (2024). Advances and future perspectives of intranasal drug delivery: A scientometric review. J. Control. Release.

[57] Keller L.-A., Merkel O., Popp A. (2022). Intranasal drug delivery: Opportunities and toxicologic challenges during drug development. Drug Deliv. Transl. Res..

[58] Rabiee N., Ahmadi S., Afshari R., Khalaji S., Rabiee M., Bagherzadeh M., Fatahi Y., Dinarvand R., Tahriri M., Tayebi L. (2020). Polymeric Nanoparticles for Nasal Drug Delivery to the Brain: Relevance to Alzheimer’s Disease. Adv. Ther..

[59] Luo D., Ni X., Yang H., Feng L., Chen Z., Bai L. (2024). A comprehensive review of advanced nasal delivery: Specially insulin and calcitonin. Euro. J. Pharm. Sci..

